# Benefits of statin therapy within a year after kidney transplantation

**DOI:** 10.1038/s41598-024-52513-6

**Published:** 2024-01-23

**Authors:** Seung Hyuk Yim, Hyun Jeong Kim, Han Ro, Jung-Hwa Ryu, Myung-Gyu Kim, Jae Berm Park, Chan-Duck Kim, Seungyeup Han, Sik Lee, Jaesok Yang, Kyu Ha Huh, Myoung Soo Kim, Juhan Lee

**Affiliations:** 1 Department of Surgery, The Research Institute for Transplantation, Yonsei University College of Medicine, Seoul, South Korea; 2 Department of Internal Medicine, Gachon University College of Medicine, Incheon, South Korea; 3 Department of Internal Medicine, Ewha Womans University Seoul Hospital, Seoul, South Korea; 4 Department of Internal Medicine, Korea University College of Medicine, Seoul, South Korea; 5 Department of Surgery, Seoul Samsung Medical Center, Sungkyunkwan University, Seoul, South Korea; 6 Department of Internal Medicine, Kyungpook National University Hospital, Daegu, South Korea; 7 Department of Internal Medicine, Dongsan Medical Center, Keimyung University, Daegu, South Korea; 8 Department of Internal Medicine, Chonbuk National University Hospital, Jeonju, South Korea; 9 Department of Internal Medicine, Yonsei University College of Medicine, Seoul, South Korea

**Keywords:** End-stage renal disease, Allotransplantation

## Abstract

Cardiovascular disease remains a leading cause of morbidity and mortality after kidney transplantation (KT). Although statins reduce cardiovascular risk and have renal benefits in the general population, their effects on KT recipients are not well-established. We studied the effects of early statin use (within 1-year post-transplantation) on long-term outcomes in 714 KT recipients from the Korean cohort study for outcome in patients with KT. Compared with the control group, statin group recipients were significantly older, had a higher body mass index, and had a higher prevalence of diabetes mellitus. During a median follow-up of 85 months, 74 graft losses occurred (54 death-censored graft losses and 20 deaths). Early statin use was independently associated with lower mortality (hazard ratio, 0.280; 95% confidence interval 0.111–0.703) and lower death-censored graft loss (hazard ratio, 0.350; 95% confidence interval 0.198–0.616). Statin therapy significantly reduced low-density lipoprotein cholesterol levels but did not decrease the risk of major adverse cardiovascular events. Biopsy-proven rejection and graft renal function were not significantly different between statin and control groups. Our findings suggest that early statin use is an effective strategy for reducing low-density lipoprotein cholesterol and improving patient and graft survival after KT.

## Introduction

Cardiovascular disease (CVD) remains a major cause of death and morbidity among kidney transplantation (KT) recipients^1,2^. While KT reduces the risk of CVD, compared to remaining on dialysis, KT recipients continue to have a significantly higher risk of CVD than the general population^3,4^. In addition to the presence of pre-existing risk factors for CVD, KT recipients are exposed to additional risks associated with the use of immunosuppressive agents^5,6^. Immunosuppressive agents can lead to metabolic changes that increase the risk of dyslipidemia, which is a major risk factor for CVD^7,8^.

Dyslipidemia is a common complication following KT^9^. In the general population, the association between dyslipidemia and an increased risk of CVD is well-documented and reducing low-density lipoprotein (LDL)-cholesterol levels has been demonstrated to significantly lower morbidity and mortality risks^10^. Statins are widely used as first-line therapy for dyslipidemia, as they effectively lower LDL-cholesterol^11^. The benefits of statin therapy are well-established in patients with chronic kidney disease (CKD), with the exception of patients undergoing dialysis^12–14^. Although current international guidelines recommend statin therapy for adult KT recipients, the efficacy of statins in these patients has not been conclusively demonstrated^15^. Furthermore, prior studies have been conducted in KT recipients treated with older immunosuppressive agents, such as cyclosporine and azathioprine, and there is a lack of research evaluating the clinical effects of statins in patients treated with modern immunosuppressive regimens^16–18^.

As KT candidates are becoming older and more likely to have diabetes mellitus, managing cardiovascular complications after KT has become increasingly important^19^. Thus, there is an urgent need to investigate the effects of statin therapy in this patient population. This study aimed to evaluate the association of early statin use with post-transplantation outcomes in KT recipients.

## Results

### Baseline characteristics

A total of 714 patients were included in this study. Their baseline characteristics are summarized in Table 1. Overall, the mean age of recipients was 45.6 ± 11.8 years, 593 (83.1%) received a kidney from a living donor, and 182 (25.5%) had diabetes mellitus at the time of transplantation. The donors had a mean age of 44.2 ± 11.9 years and a mean estimated Glomerular Filtration Rate (eGFR) of 90.9 ± 27.6 mL/min/1.73 m^2^. Ninety donors (12.6%) had hypertension. Pretransplant donor-specific antibodies were identified in 48 recipients (6.7%), and 140 recipients (19.6%) underwent ABO-incompatible KT.

**Table 1:** Baseline characteristics according to statin therapy.

	Statin (n = 414)	Control (n = 300)	p-value
Recipients			
Age, years	47.2 ± 10.7	43.4 ± 13.0	< 0.001
Male, *n* (%)	254 (61.4)	204 (68.0)	0.080
Dialysis vintage, months	23.0 ± 37.7	26.1 ± 44.1	0.341
Donor-specific antibodies, *n* (%)	29 (7.0)	19 (6.3)	0.840
ABO incompatible KT, *n* (%)	76 (18.4)	64 (21.3)	0.372
Donors			
Deceased donor, *n* (%)	68 (16.4)	53 (17.7)	0.737
Donor age, years	43.9 ± 11.7	44.6 ± 12.2	0.407
Male donor, *n* (%)	206 (49.8)	137 (45.7)	0.315
eGFR, mL/min/1.73m^2^	90.7 ± 27.0	91.1 ± 28.4	0.848
Hypertension, *n* (%)	50 (12.1)	40 (13.3)	0.700
Diabetes mellitus, *n* (%)	16 (3.9)	6 (2.0)	0.229
Systolic blood pressure, mmHg	122.2 ± 15.7	119.9 ± 18.6	0.077
Body mass index, kg/m^2^	23.8 ± 3.1	23.8 ± 3.0	0.981
Recipient cardiovascular risk factors			
Body mass index, kg/m^2^	23.3 ± 3.6	22.1 ± 3.3	< 0.001
Hypertension, *n* (%)	385 (93.0)	275 (91.7)	0.604
Waist-hip ratio	0.9 ± 0.1	0.9 ± 0.1	0.686
Diabetes mellitus, *n* (%)	118 (28.5)	64 (21.3)	0.037
MACE prior to KT, *n* (%)	43 (10.4)	20 (6.7)	0.113
Statin use prior to KT, *n* (%)	173 (41.8)	50 (16.7)	< 0.001
LDL-cholesterol, mg/dL	88.6 ± 33.1	76.5 ± 28.1	< 0.001
HDL-cholesterol, mg/dL	44.9 ± 15.6	46.0 ± 17.7	0.383
Total cholesterol, mg/dL	163.0 ± 43.4	144.4 ± 38.6	< 0.001
Recipient smoking, *n* (%)			0.743
Current	26 (6.3)	21 (7.0)	
Former	170 (41.1%)	115 (38.3)	
Systolic blood pressure, mmHg	138.1 ± 18.5	135.7 ± 19.1	0.081

Data are presented as number (percentage) or mean ± standard deviation.

*eGFR* estimated glomerular filtration rate, *HDL* high-density lipoprotein, *MACE* major adverse cardiovascular event, *KT* kidney transplantation, *LDL* low-density lipoprotein.

There were several significant differences in recipient characteristics between the statin and control groups. Statin group KT recipients were significantly older than control group recipients (47.2 ± 10.7 years versus 43.4 ± 13.0 years, *p* < 0.001). Regarding CVD risk factors, recipients in the statin group had a higher body mass index (23.3 ± 3.6 kg/m^2^ versus 22.1 ± 3.3 kg/m^2^, *p* < 0.001), higher prevalence of pretransplant diabetes mellitus (28.5% versus 21.3%, *p* = 0.037), and higher prevalence of pretransplant statin use (42.1% versus 15.9%, *p* < 0.001), compared to the control group. Despite their more frequent pretransplant statin use, the statin group patients had significantly higher baseline LDL-cholesterol and total cholesterol levels than the control group (*p* < 0.001). There were no significant differences in high-density lipoprotein cholesterol levels or smoking history between the two groups. The mean systolic blood pressure at the time of transplantation was slightly higher in the statin group, but the difference between groups was not statistically significant.

### Immunosuppression

Table 2 summarizes the immunosuppressants utilized in our study cohort. Of the 714 patients, 712 (99.7%) received induction therapy, which consisted of basiliximab in 642 patients (90%) and anti-thymocyte globulin in 70 patients (9.8%). For maintenance immunosuppression, 676 patients (94.7%) received tacrolimus, and the majority of patients also were treated with prednisolone (87.8%) and mycophenolate mofetil (MMF) (83.5%). A total of 51 patients (7.1%) received a mammalian target of rapamycin (mTOR) inhibitor, and the proportion of patients who received mTOR inhibitor was significantly higher in the statin group than in the control group. Tacrolimus trough levels were consistently lower in the statin group than in the control group at all times throughout the follow-up period, except at the first-year post-transplantation. The MMF dose did not differ significantly between statin and control groups.

**Table 2:** Immunosuppression.

	Statin (*n* = 414)	Control (*n* = *300*)	*p*-value
Induction			0.650
Basiliximab, *n* (%)	380 (91.8)	262 (87.3)	
Anti-thymocyte globulin, *n* (%)	38 (9.2)	32 (10.7)	
Maintenance			
Calcineurin inhibitor, *n* (%)			0.022
Tacrolimus	389 (94.0)	287 (95.7)	
Cyclosporine	29 (7.0)	9 (3.0)	
Prednisolone, *n* (%)	394 (95.2)	233 (77.7)	< 0.001
Mycophenolate mofetil, *n* (%)	345 (83.3)	251 (83.7)	0.423
mTOR inhibitor, *n* (%)	44 (10.6)	7 (2.3)	< 0.001
Tacrolimus trough level			
1-year, ng/mL	5.8 ± 2.3	6.1 ± 2.1	0.084
2-year, ng/mL	5.3 ± 2.1	5.7 ± 1.9	0.011
3-year, ng/mL	5.2 ± 1.8	5.8 ± 1.9	< 0.001
4-year, ng/mL	5.2 ± 1.9	5.6 ± 2.1	0.024
5-year, ng/mL	5.1 ± 1.8	5.7 ± 2.0	0.002
6-year, ng/mL	5.1 ± 1.9	5.7 ± 2.0	0.007
7-year, ng/mL	5.1 ± 1.9	5.6 ± 2.2	0.036

Data are presented as number (percentage) or mean ± standard deviation.

*mTOR* mammalian target of rapamycin.

### Graft and patient survival

During follow-up, there were 74 graft losses: 54 death-censored graft losses and 20 patient deaths. The causes of graft loss are described in Supplement 1. The predominant cause identified was the rejection (66.7%), followed by primary graft failure (11.1%). Comparative analysis between the statin group and the control group revealed no significant differences in graft loss rates (*p* = 0.485).

**Figure 1:**
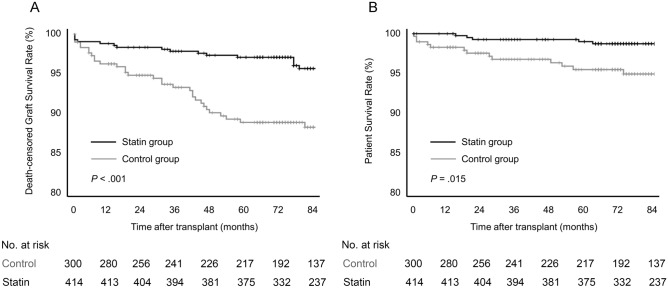
Kaplan–Meier curves for (**A**) death-censored graft survival and (**B**) patient survival by early statin use.

As shown in Fig. 1A, the death-censored graft survival rate was significantly higher in the statin group than in the control group (*p* < 0.001). The 1-year, 3-year, and 5-year death-censored graft survival rates were 99.8%, 98.8%, and 98.0% for the statin group and 95.0%, 92.0%, and 87.7% for the control group. Multivariable Cox regression analysis revealed that early statin use was independently associated with lower death-censored graft loss (adjusted hazard ratio [aHR], 0.231; 95% confidence interval CI 0.126–0.424; *p* < 0.001; Table 3). Beside early statin use, other independent factors associated with graft loss were the history of MACE prior to KT (aHR, 2.222; 95% CI 1.067–4.627), desensitization (aHR, 2.212; 95% CI 1.167–4.192), female donor (aHR, 2.383; 95% CI 1.246–4.558), and donor hypertension (aHR, 2.316; 95% CI 1.141–4.700).

**Table 3:** Risk factors for death-censored graft loss.

Factors	Univariable		Multivariable	
	cHR (95% CI)	*p*-value	aHR (95% CI)	*p*-value
Recipient age	0.999 (0.976–1.022)	0.903		
Male	1.037 (0.593–1.813)	0.899		
Diabetes mellitus	1.202 (0.663–2.181)	0.544		
Dialysis vintage	1.002 (0.996–1.008)	0.427		
Smoking history (including former smoker)	0.570 (0.139–2.34)	0.435		
Systolic blood pressure (per 1 mmHg)	1.014 (1.000–1.027)	0.051	1.013 (1.000–1.027)	0.050
MACE prior to KT	1.671 (0.817–3.419)	0.159	2.222 (1.067–4.627)	0.033
Statin use prior to KT	0.787 (0.428–1.446)	0.440		
Desensitization	1.556 (0.876–2.765)	0.131	2.212 (1.167–4.192)	0.015
Early statin use	0.241 (0.133–0.438)	< 0.001	0.231 (0.126–0.424)	< 0.001
Living donor	0.435 (0.245–0.773)	0.005	0.674 (0.293–1.547)	0.352
Female donor	0.556 (0.318–0.972)	0.039	2.383 (1.246–4.558)	0.009
Donor age	1.020 (0.997–1.044)	0.093	0.991 (0.964–1.019)	0.525
Donor hypertension	3.298 (1.838–5.916)	< 0.001	2.316 (1.141–4.700)	0.020
Donor eGFR	0.991 (0.982–1.000)	0.041	0.988 (0.976–1.000)	0.060

*aHR* adjusted hazard ratio, *cHR* crude hazard ratio, *CI* confidence interval, *eGFR* estimated glomerular filtration rate, *MACE* major adverse cardiovascular event, *KT* kidney transplantation.

Overall patient survival rate was significantly higher in the statin group than in the control group (*p* = 0.015; Fig. 1B). The 1-year, 3-year, and 5-year overall patient survival rates were100.0%, 99.3%, and 99.0% for the statin group and 96.9%, 95.1% and 93.4% for the control group. Early statin use was independently associated with lower all-cause mortality in multivariable Cox regression analysis (aHR, 0.280; 95% CI 0.111–0.704; *p* = 0.007).

### LDL-cholesterol levels and major adverse cardiovascular events (MACE)

**Figure 2:**
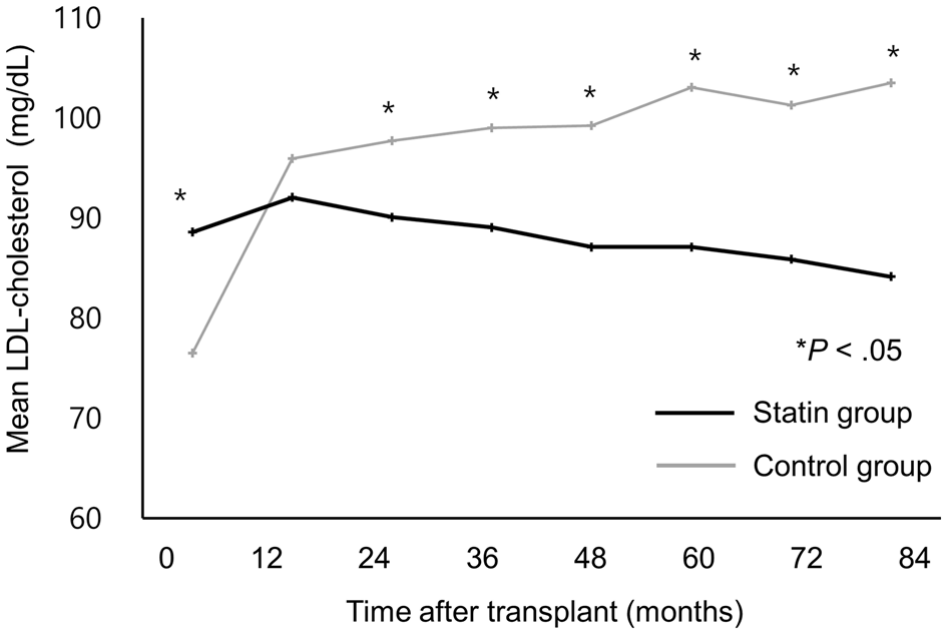
Serum low-density lipoprotein cholesterol levels over time by early statin use after kidney transplantation.

Changes in LDL-cholesterol levels after transplantation are shown in Fig. 2. In the statin group, LDL-cholesterol levels increased slightly during the first year, followed by a continuous downward trend after 2 years; the levels remained below 100 mg/dL during the entire study period. In the control group, LDL-cholesterol levels increased sharply during the first year after transplantation, after which they continued to trend upward for the rest of the follow-up period. LDL-cholesterol levels were consistently and significantly lower in the statin group than in the control group beginning at 2 years after post-transplantation.

During follow-up, MACE occurred in 37 patients. The crude incidence of MACE was significantly higher in the statin group than in the control group (*p* = 0.010), but this difference was no longer significant after adjusting for other variables (aHR, 1.718; 95% CI 0.730–4.042). Recipient age (aHR, 1.082; 95% CI 1.038–1.128), pretransplant diabetes mellitus (aHR, 3.923; 95% CI 1.869–8.232), and higher baseline LDL-cholesterol level (aHR, 1.010; 95% CI 1.001–1.019) were independent risk factors for MACE.

### Biopsy-proven acute rejection and graft renal function

A total of 101 biopsy-proven acute rejection episodes, including 70 T-cell mediated Rejections (TCMR) and 31 Antibody Mediated Rejections (AMR) occurred among 91 recipients. The cumulative incidence of biopsy-proven acute rejection was 9% in the statin group and 10% in the control group, with no significant difference between groups (*p* = 0.693). There was also no significant difference between groups when the cumulative incidence of AMR or TCMR was analyzed separately.

**Figure 3:**
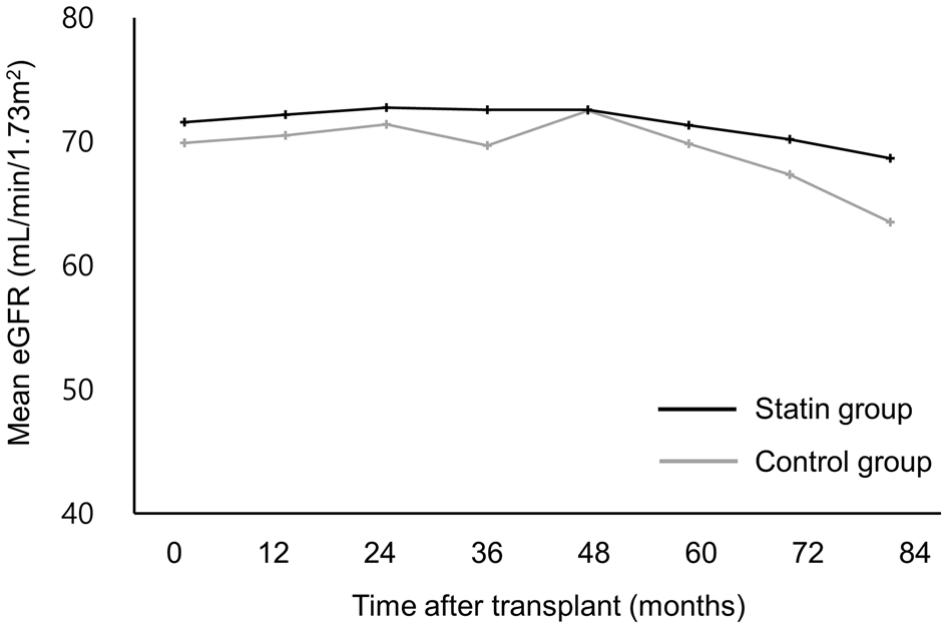
Estimated glomerular filtration rates over time in the statin and control groups.

As shown in Fig. 3, mean eGFRs after KT were not significantly different between statin and control groups throughout the study period. Mean eGFR at 5 years after KT was 72.6 ± 21.0 mL/min/1.73 m^2^ in the statin group and 72.5 ± 20.4 mL/min/1.73 m^2^ in the control group.

### Sensitivity analysis

We conducted a sensitivity analysis on this study results employing several methods. Firstly, we validated the outcomes of death censored graft survival and overall survival exclusively for the group of patients using tacrolimus, by excluding patients who did not use tacrolimus (n = 38). Additionally, we confirmed the impact of early statin use on the study’s outcome through multivariable Cox regression analysis, which demonstrated that early statin use was independently associated with the outcome (Supplements 2, 3). Secondly, we ensured the robustness of our findings by excluding patients with graft loss within one year after transplantation (n = 21). By doing so, we verified that the use of statin was remained consistent even when focusing on patients with longer-term survival (Supplements 4,5). Thirdly, we extended the standard definition of early statin use to include patients who initiated statin therapy within two years after the transplant, including an additional 78 patients. This extension allowed us to examine if the results would hold when considering a broader time frame for statin initiation (Supplements 6,7). Fourthly, we included 242 patients who either discontinued statin therapy during the study or started more than a year after transplantation. It was confirmed that early statin use remained an independent protective factor for both graft and patient survival (Supplements 8, 9). Lastly, patients with prior history of MACE to KT were excluded: 43 from the statin group and 20 from the control group. We found that early statin use continued to be an independently associated with both graft and patient survival (Supplements 10, 11).

## Discussion

In this large multicenter cohort study, we investigated the clinical outcomes associated with early statin therapy initiated within 1-year following KT. Our results showed that early statin use was associated with significant improvements in death-censored graft survival and patient survival, even after adjusting for potential confounding factors. In addition, we observed a significant reduction in LDL-cholesterol levels among KT recipients treated with statins. Nevertheless, early statin use was not associated with a reduced risk of MACE. There was also no statistically significant difference in the incidence of biopsy-proven acute rejection between groups, despite consistently lower tacrolimus trough levels during follow-up in the statin group.

Statin therapy effectively reduces LDL-cholesterol, prevents MACE, and improves patient survival in the general population^10^. Benefits of statin therapy have also been observed in patients with varying degrees of CKD, who are at increased risk of CVD^13,14^. Current guidelines recommend the use of statins in KT recipients, but limited clinical evidence supports the efficacy of statin therapy in this population^15^. The only large-scale clinical trial investigating the effect of statin therapy in KT recipients, the Assessment of Lescol in Renal Transplantation (ALERT) trial, was conducted two decades ago and used older immunosuppressive agents, such as cyclosporine and azathioprine^16^. Given the increasing age and prevalence of MACE in KT candidates, it is crucial to investigate the current clinical relevance of statin therapy in KT recipients. To the best of our knowledge, this is the first large-scale multicenter cohort study to evaluate the effects of statins on transplant outcomes among KT recipients treated with current immunosuppressive agents.

In the present study, early statin use was associated with improved death-censored graft survival, as well as patient survival. This finding remained consistent, even after adjusting for several confounding factors, including donor and recipient characteristics, immunologic factors, and immunosuppression. Among these aspects, a history of major adverse cardiac events (MACE) before kidney transplantation (KT), desensitization procedures, the use of female donors, and hypertension in donors were all identified as independent indicators of graft failure. These results are in agreement with those obtained from earlier studies.

While the exact mechanism of survival benefits from early statin use cannot be determined in a retrospective study, renoprotective effects of statins may be a contributing factor. Renoprotective effects of statins vary according to the type and dose of statin. Compared to other statins, atorvastatin has been reported to have greater renoprotective effects, which occur in a dose‐dependent manner^20,21^. Nevertheless, previous studies in KT recipients have not shown significant associations between statin therapy and renal outcomes. In the ALERT trial, use of fluvastatin 40–80 mg/day for an average of 5 years after KT was not associated with improved renal outcomes^16,22^. Previous retrospective studies had limitations, such as the statin groups including patients who underwent transplantation under unfavorable donor conditions (e.g. advanced age) or who discontinued statin therapy^17,18^.

Our data confirmed a significant association between early statin use after KT and reduced LDL-cholesterol levels. However, the crude incidence of MACE was higher in the statin group than in the control group. As with other retrospective studies, KT recipients in our study were not randomly allocated to statin therapy. The statin group had a higher prevalence of comorbidities associated with an increased risk of CVD, including diabetes mellitus, advanced age, and obesity^6^. After adjusting for confounding factors, we observed no significant difference in the incidence of MACE between the statin and control groups. The lack of a significant reduction in the incidence of MACE despite reduced LDL-cholesterol levels with statin therapy may be at least partly attributed to the relatively low overall incidence of MACE in our cohort. The incidence of MACE was lower in our patients than in previous study populations, possibly because of ethnic differences (our study included mostly East Asians) and a high proportion of living-donor KTs in our patients^23,24^. To confirm the effects of statin therapy on MACE in KT recipients, future larger-scale studies are necessary.

Beyond cholesterol-lowering effects, statins have other pleiotropic effects, including immune modulation^25,26^. Statins inhibit the mevalonate pathway, which plays a critical role in immune cell activation, migration, proliferation, and cytokine production^27^. In line with in vitro results, earlier studies showed significant reductions in acute rejection after solid organ transplantation with statin therapy^26,28,29^. However, subsequent randomized studies failed to show an association between statin use and acute rejection^30,31^. Most previous studies were conducted in KT recipients receiving cyclosporine, without contemporary immunologic or histologic assessment^28,30,31^. Our data revealed no statistically significant difference in the incidence of biopsy-proven acute rejection between groups, despite the statin group having consistently lower tacrolimus trough concentrations during follow-up. Whether statin therapy could be used as a tacrolimus-sparing agent in KT recipients is an important issue but beyond the scope of this study. Our findings highlight the need for future research regarding the pleiotropic effects of statins in KT recipients.

The present study has limitations. First, as with all observational studies, our results may be subject to confounding by indication. To address this issue, we attempted to minimize potential confounding by adjusting for various donor, recipient, and immunologic factors. Second, the high proportion of living donors and the predominantly East Asian population in our study cohort may limit the generalizability of our findings to other populations. Third, data regarding the type and dose of statins were not collected. However, unlike other observational studies, we tracked the prescription of statins annually, providing a more robust assessment of the effects of statin therapy.

In conclusion, our study demonstrated that early statin therapy following KT effectively lowers LDL-cholesterol levels and is associated with improved graft and patient survival. These benefits were consistently observed across patients with varying characteristics. Furthermore, despite lower tacrolimus trough concentrations in patients receiving early statin therapy, there was no significant difference in the incidence of biopsy-proven acute rejection between statin and control groups.

## Methods

### Study population

**Figure 4:**
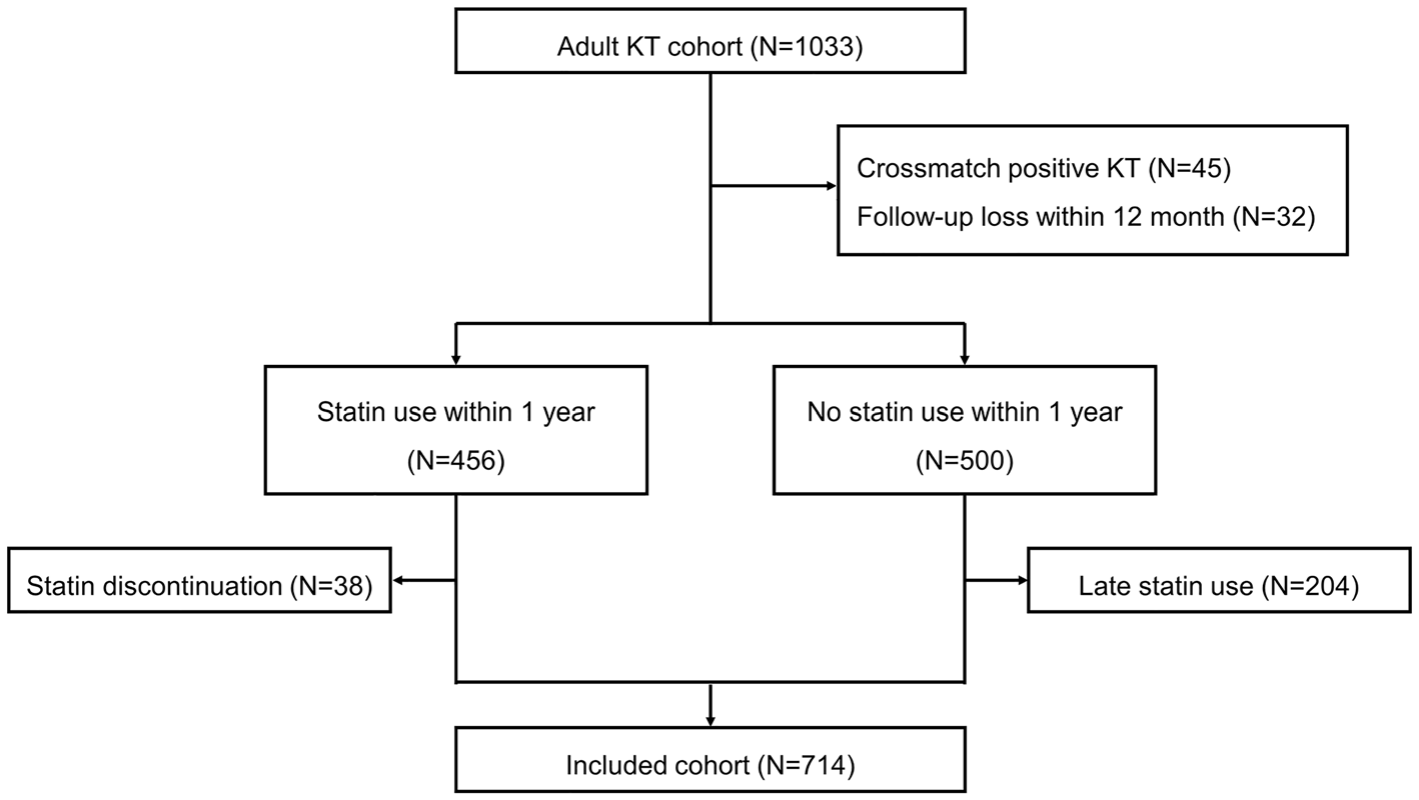
Study flowchart.

The Korean Cohort Study for Outcomes in Patients with KT (KNOW-KT) is a multicenter, prospective, observational cohort study of adult KT recipients from eight university-affiliated Korean transplantation centers (ClinicalTrials.gov number, NCT02042963). The detailed study design and methods of KNOW-KT have been described previously^32^. Between 2012 and 2016, we screened 1080 KT recipients enrolled in KNOW-KT. Patients who underwent a crossmatch positive KT, were lost to follow-up within 12 months, or lacked sufficient data were excluded. The patients were grouped into two groups: those who received statins within 1-year after KT (“statin group”) and those who did not (“control group”). For the final analysis, we further excluded patients who discontinued statin therapy during the study period or who initiated statins more than 1-year after transplantation (Fig. 4).

The KNOW-KT study protocol was approved by the Institutional Review Board of each participating center (Yonsei University Severance Hospital, Seoul National University Hospital, Sungkyunkwan University Samsung Medical Center, Korea University Anam Hospital, Kyungpook National University Hospital, Chonbuk National University Hospital, Gachon University Gil Hospital, and Keimyung University Dongsan Medical Center), and all participants provided written informed consent prior to enrollment. All research procedures were conducted in accordance with the Declaration of Helsinki and were approved by the Institutional Review Board of Severance Hospital (4-2022-0953). All clinical and research activities were consistent with the Principles of the Declaration of Istanbul, as outlined in the Declaration of Istanbul on Organ Trafficking and Transplant Tourism.

### Data collection and measurements

Baseline demographic and medical history data, including age, sex, smoking history, history of comorbidities, and medications, were recorded at the time of enrollment. Anthropometric data, including height, weight, and waist-to-hip ratio, were also collected. Laboratory measurements at baseline included hemoglobin and serum levels of creatinine, total cholesterol, LDL-cholesterol, high-density lipoprotein cholesterol, triglycerides, uric acid, albumin, calcium, and phosphorus. Serum creatinine was measured using an isotope‐dilution mass spectrometry‐tractable method, and the eGFR was calculated using the chronic kidney disease epidemiology collaboration equation^33^. All of these routine laboratory assessments were performed by the local laboratory.

We collected data on routine laboratory values and medication usage annually throughout the study period, including information regarding the medication start date, discontinuation date, and duration of therapy. Additional information on medication usage, including the use of statins, was collected for patients who experienced death or graft loss within one year. Participants were regularly followed according to the study protocol, and any events related to the study outcomes during the follow-up period were recorded.

### Exposure

The exposure variable of interest was early statin use, defined as statin therapy initiated within 1-year after KT. Based on annual medication data, patients who initiated statin therapy after 1-year (i.e. late use) or who discontinued statin therapy during the follow-up period were excluded from the study. The control group consisted of recipients who did not use statins after transplantation, irrespective of their history of statin use before transplantation.

### Study endpoints and definitions

The primary endpoints were graft survival and patient survival. Death-censored graft loss was defined as a return to long-term dialysis or re-transplantation. Graft survival was calculated from the date of transplantation to the date of graft loss or December 31, 2021 (the end of the study follow-up period). For patients who died with a functioning graft, graft survival was censored at the time of death. Patient survival was evaluated from the date of transplantation to the date of death or last follow-up. The secondary study endpoints included serum LDL- cholesterol levels, MACE, biopsy-proven acute rejection, and graft renal function. MACE was defined as myocardial infarction, stroke, or new onset or aggravation of congestive heart failure. All acute rejections were confirmed by biopsy and classified by local pathologists as TCMR or AMR, according to the most recent Banff criteria at the time of biopsy^34^.

### Immunosuppression

Immunosuppressive agents were administered in accordance with the standard protocol at each center. Most patients received induction immunosuppression with basiliximab (20 mg on days 0 and 4 post-transplantation) or anti-thymocyte globulin (1.5 mg/kg per day for the first 4 days after transplantation). Maintenance immunosuppression consisted of a calcineurin inhibitor (tacrolimus or cyclosporine), prednisolone, and MMF or mTOR inhibitor. The target trough concentration for tacrolimus was 5–12 ng/mL. The initial dose of intravenous methylprednisolone was 500–1000 mg, which was gradually reduced until treatment was converted to oral prednisolone (5–10 mg/day).

### Statistical analysis

Depending on the type of variable, data were expressed as frequency, mean ± standard deviation, or median and interquartile range. The chi-square test or Fisher’s exact test was used as appropriate to compare categorical variables. Continuous variables were compared using Student’s *t* test for parametric data or Mann–Whitney test for nonparametric data. Kaplan–Meier curves and the log-rank test were used to compare patient and graft survival between groups. Cox proportional hazard regression models were used to evaluate the association between early statin use and time-to-event outcomes. Clinically important variables and variables with a *p*-value ≤ 0.2 in univariable analyses were introduced into multivariable regression models. The sensitivity analysis was conducted to validate the results obtained from this study. Various inclusion criteria were employed to assess the robustness and reliability of our findings in death-censored graft survival and patient survival. All tests were two-tailed, and *p*-values < 0.05 were considered statistically significant. Statistical analyses were performed using SPSS software (version 26.0; IBM Corp., Armonk, NY, USA).

### Supplementary Information

Supplementary Information.

## Data Availability

The data underlying this article will be shared on reasonable request to the corresponding author.
