# Supplementary material for: Benefits of statin therapy within a year after kidney transplantation
Source: Sci Rep. 2024 Jan 23;14:2002. doi: 10.1038/s41598-024-52513-6 (PMC10805738; doi:10.1038/s41598-024-52513-6)
Supplement: Supplementary file 1 — Supplementary Information. [file 41598_2024_52513_MOESM1_ESM.docx]

**Supplemental Material**

**Supplement 1.** Causes of graft loss

**Supplement 2.** Kaplan-Meier curves for (A) death-censored graft survival and (B) patient survival by early statin use, excluding patients who did not use tacrolimus.

**Supplement 3.** Risk factors for (A) death-censored graft survival and (B) patient survival by early statin use, excluding patients who did not use tacrolimus.

**Supplement 4.** Kaplan-Meier curves for (A) death-censored graft survival and (B) patient survival by early statin use, excluding patients with graft loss within one year.

**Supplement 5.** Risk factors for (A) death-censored graft survival and (B) patient survival by early statin use, excluding patients with graft loss within one year.

**Supplement 6.** Kaplan-Meier curves for (A) death-censored graft survival and (B) patient survival by early statin use, extending the initiation time of statin treatment from 1-year to 2-years.

**Supplement 7.** Risk factors for (A) death-censored graft survival and (B) patient survival by early statin use, extending the initiation time of statin treatment from 1-year to 2-years.

**Supplement 8.** Kaplan-Meier curves for (A) death-censored graft survival and (B) patient survival by early statin use, including patients who discontinued statin therapy during the study period or who initiated statins more than 1-year after transplantation.

**Supplement 9.** Risk factors for (A) death-censored graft survival and (B) patient survival by early statin use, including patients who discontinued statin therapy during the study period or who initiated statins more than 1-year after transplantation.

**Supplement 1. Causes of graft loss**

| **Cause of graft loss** | **Statin (n=15)** | **Control (n=39)** | **Total (n=54)** |
| --- | --- | --- | --- |
| *Rejection* | 13 (86.7) | 23 (59.0) | 36 (66.7) |
| *BK virus nephropathy* | 0 (0.0) | 3 (7.7) | 3 (5.6) |
| *Poor compliance* | 0 (0.0) | 2 (5.1) | 2 (3.7) |
| *Postoperative complication* | 0 (0.0) | 2 (5.1) | 2 (3.7) |
| *Primary graft failure* | 1 (6.7) | 5 (12.8) | 6 (11.1) |
| *Others* | 1 (6.7) | 4 (10.3) | 5 (9.3) |

Data are presented as number (percentage).

**Supplement 2.** Kaplan-Meier curves for (A) death-censored graft survival and (B) patient survival by early statin use, excluding patients who did not use tacrolimus.

**(A)**


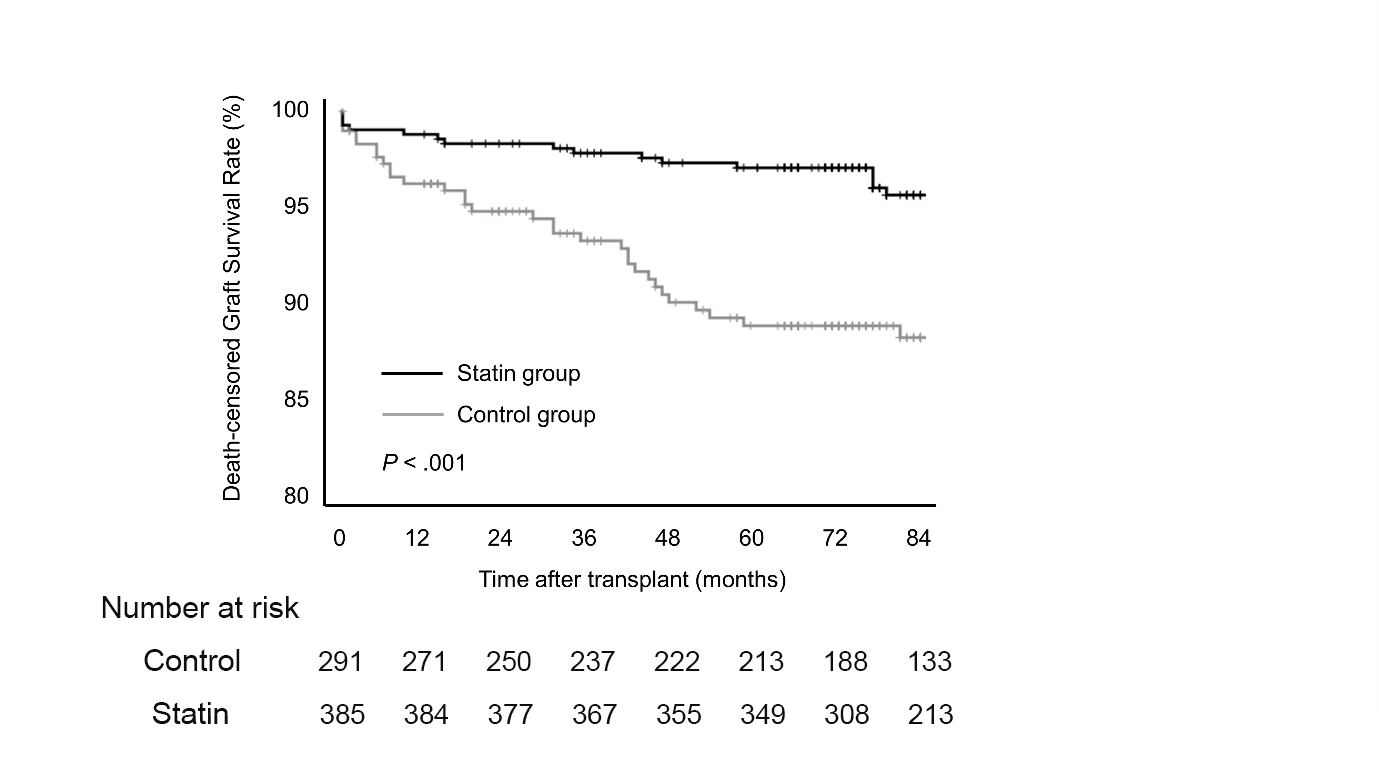


**(B)**

**
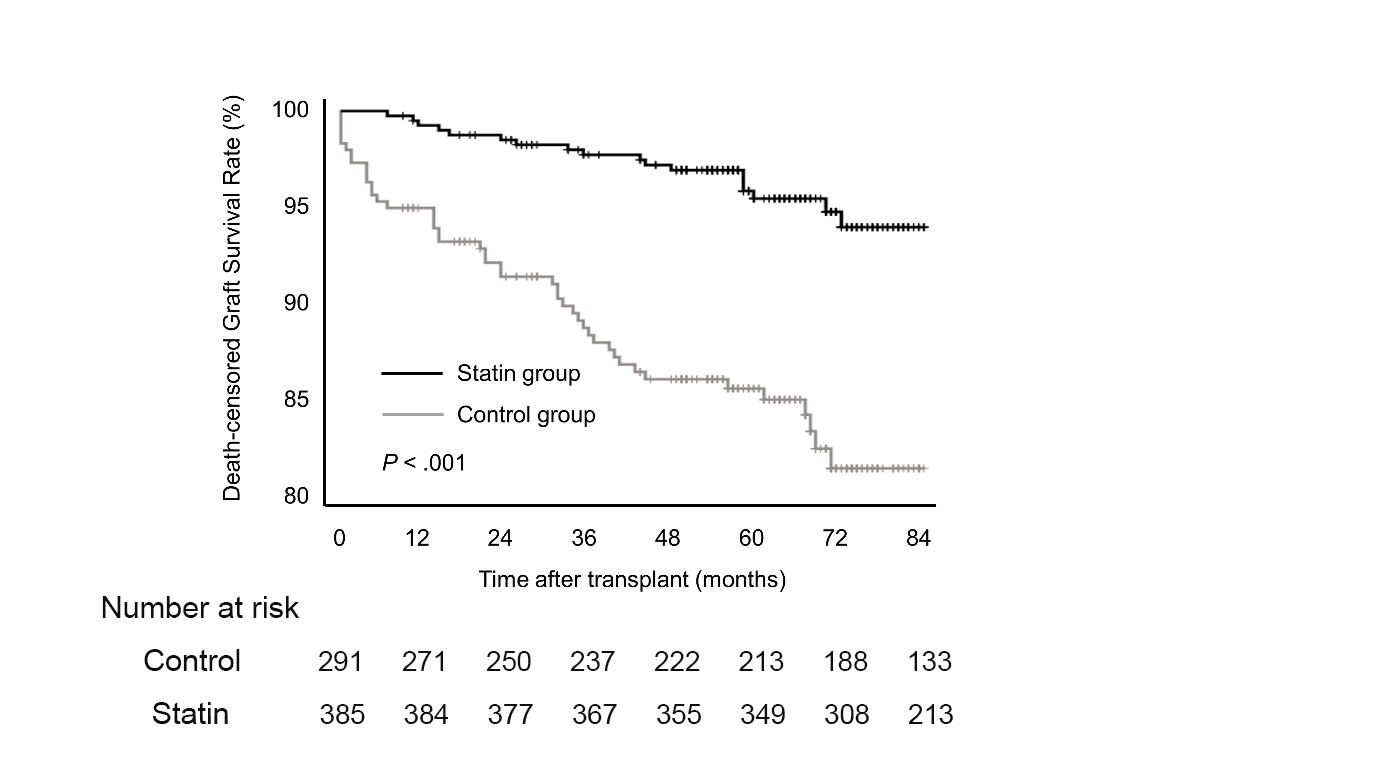
**

**Supplement 3.** Risk factors for (A) death-censored graft survival and (B) patient survival by early statin use, excluding patients who did not use tacrolimus.

**(A)**

| **Factors** | **Univariable**  **cHR (95% CI)** | **Multivariable**  **aHR (95% CI)** |
| --- | --- | --- |
| Recipient age | 1.007 (0.982-1.033) |  |
| Female | 1.478 (0.768-2.844) |  |
| Diabetes mellitus | 1.333 (0.291-6.100) |  |
| Dialysis vintage | 0.998 (0.988-1.008) |  |
| Systolic blood pressure (per 1 mmHg) | 1.008 (0.994-1.023) |  |
| Statin use prior to KT | 1.600 (0.802-3.190) |  |
| Desensitization ^†^ | 3.209 (1.587-6.487) | 2.743 (1.431-5.260) |
| Early statin use ^†^ | 0.181 (0.090-0.367) | 0.227 (0.118-0.438) |
| Deceased donor | 2.043 (0.623-6.703) |  |
| Female donor ^†^ | 2.608 (1.279-5.318) | 2.225 (1.177-4.207) |
| Donor age | 0.980 (0.951-1.009) |  |
| Donor hypertension ^†^ | 2.598 (1.207-5.595) | 2.260 (1.151-4.439) |
| Donor eGFR ^†^ | 0.988 (0.975-1.001) | 0.984 (0.974-0.994) |

aHR, adjusted hazard ratio; cHR, crude hazard ratio; CI, confidence interval; eGFR, estimated glomerular filtration rate; KT, kidney transplant.

†P value <0.05

**(B)**

| **Factors** | **Univariable**  **cHR (95% CI)** | **Multivariable**  **aHR (95% CI)** |
| --- | --- | --- |
| Recipient age | 1.019 (0.997-1.043) |  |
| Female | 1.429 (0.802-2.545) |  |
| Diabetes mellitus | 0.750 (0.168-3.346) |  |
| Dialysis vintage | 0.998 (0.990-1.007) |  |
| Systolic blood pressure (per 1 mmHg) | 1.005 (0.993-1.018) |  |
| Statin use prior to KT ^†^ | 1.723 (0.948-3.131) | 1.860 (1.032-3.352) |
| Desensitization ^†^ | 3.025 (1.638-5.587) | 2.971 (1.625-5.431) |
| Early statin use ^†^ | 0.188 (0.102-0.344) | 0.211 (0.117-0.381) |
| Deceased donor ^†^ | 3.128 (1.163-8.415) | 3.313 (1.753-6.263) |
| Female donor ^†^ | 2.425 (1.300-4.522) | 1.972 (1.151-3.377) |
| Donor age | 0.990 (0.965-1.015) |  |
| Donor hypertension ^†^ | 2.580 (1.325-5.023) | 2.662 (1.505-4.711) |
| Donor eGFR | 0.996 (0.985-1.007) |  |

aHR, adjusted hazard ratio; cHR, crude hazard ratio; CI, confidence interval; eGFR, estimated glomerular filtration rate; KT, kidney transplant.

†P value <0.05

**Supplement 4.** Kaplan-Meier curves for (A) death-censored graft survival and (B) patient survival by early statin use, excluding patients with graft loss within one year.

**(A)**

**
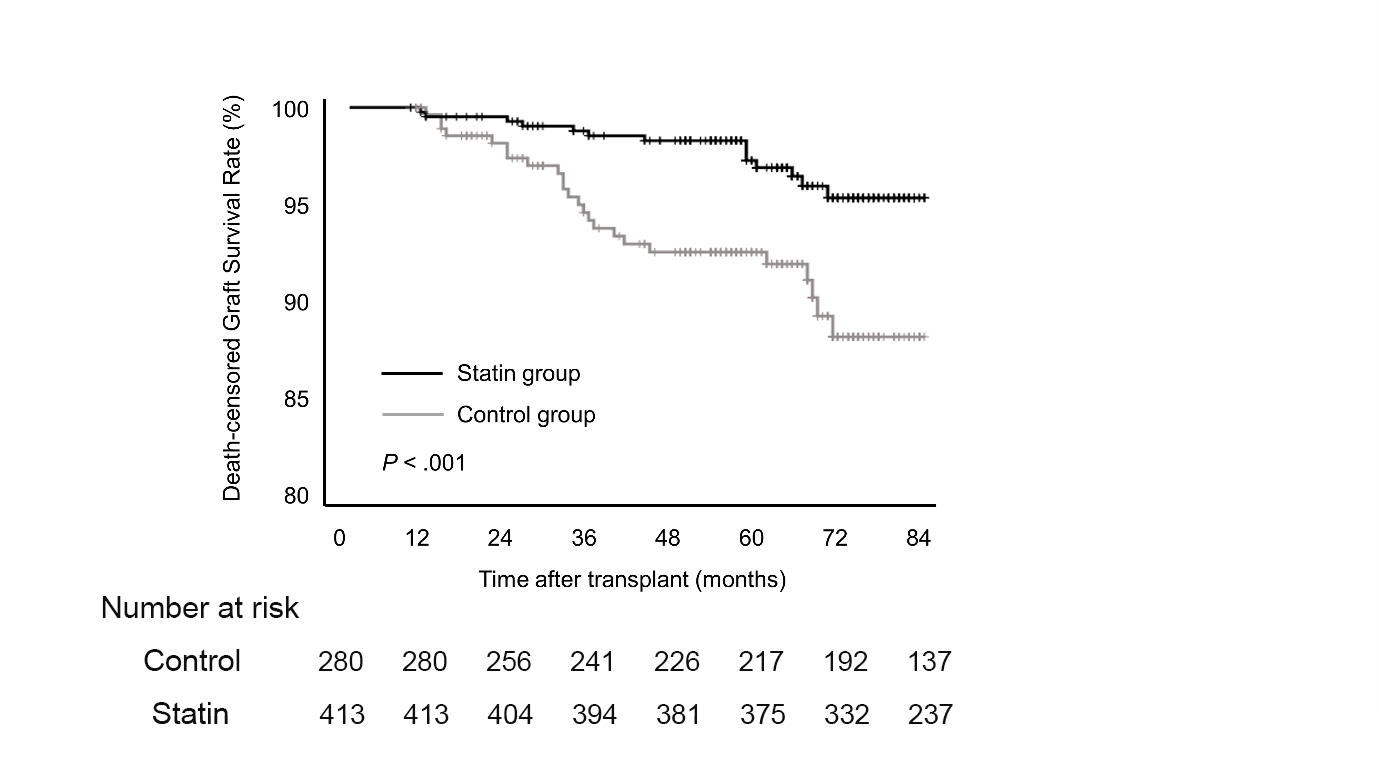
**

**(B)**

**
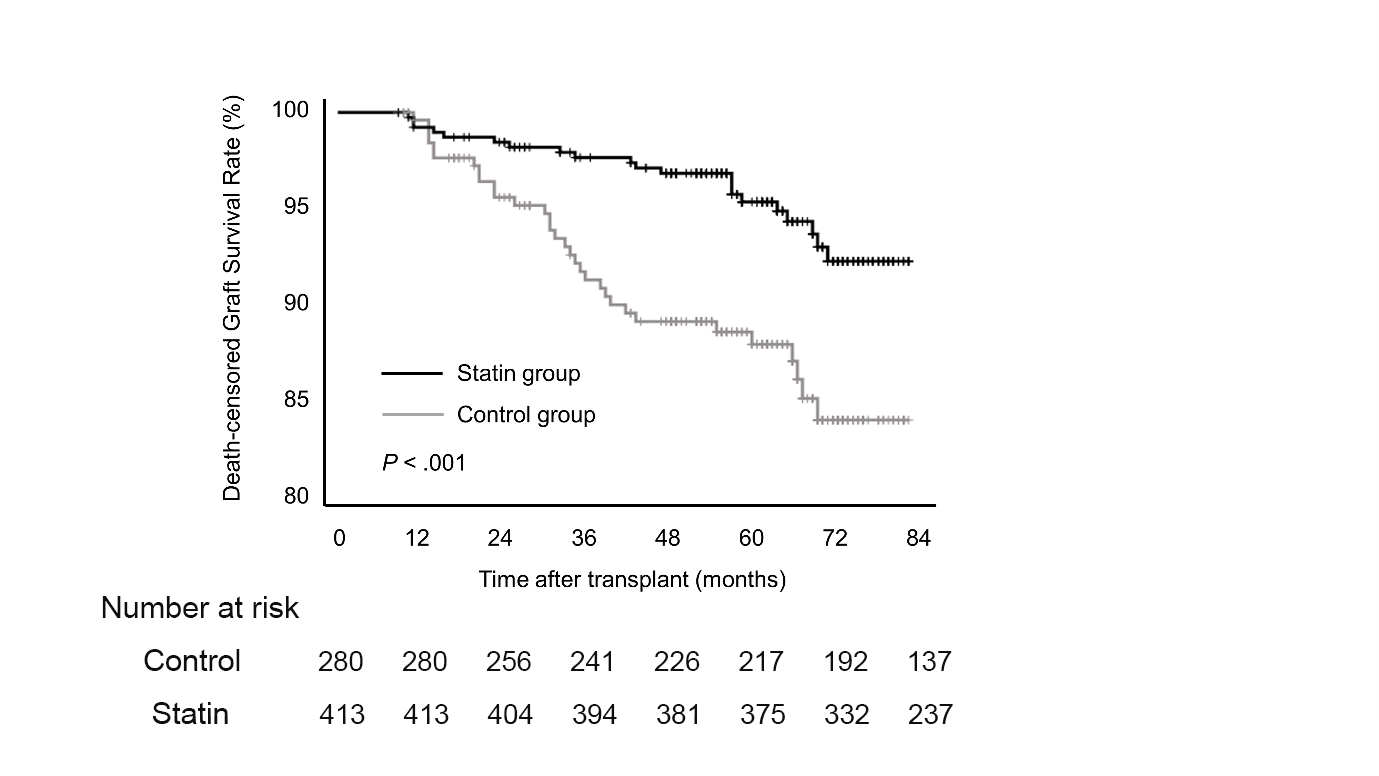
**

**Supplement 5.** Risk factors for (A) death-censored graft survival and (B) patient survival by early statin use, excluding patients with graft loss within one year.

**(A)**

| **Factors** | **Univariable**  **cHR (95% CI)** | **Multivariable**  **aHR (95% CI)** |
| --- | --- | --- |
| Recipient age | 1.000 (0.972-1.029) |  |
| Female^†^ | 2.082 (1.027-4.218) | 2.044 (1.025-4.077) |
| Diabetes mellitus | 0.939 (0.119-7.394) |  |
| Dialysis vintage | 0.995 (0.983-1.008) |  |
| Systolic blood pressure (per 1 mmHg) ^†^ | 1.016 (1.000-1.032) | 1.015 (1.000-1.031) |
| Statin use prior to KT | 0.869 (0.378-2.001) |  |
| Desensitization ^†^ | 2.197 (1.023-4.719) | 2.173 (1.042-4.534) |
| Early statin use ^†^ | 0.303 (0.147-0.621) | 0.291 (0.149-0.570) |
| Deceased donor | 1.617 (0.432-6.062) |  |
| Female donor ^†^ | 2.534 (1.144-5.615) | 2.726 (1.290-5.761) |
| Donor age | 1.006 (0.974-1.040) |  |
| Donor hypertension | 1.15 (0.437-3.025) |  |
| Donor eGFR ^†^ | 0.99 (0.975-1.006) | 0.987 (0.976-0.998) |

aHR, adjusted hazard ratio; cHR, crude hazard ratio; CI, confidence interval; eGFR, estimated glomerular filtration rate; KT, kidney transplant.

†P value <0.05

**(B)**

| **Factors** | **Univariable**  **cHR (95% CI)** | **Multivariable**  **aHR (95% CI)** |
| --- | --- | --- |
| Recipient age | 1.007 (0.983-1.033) |  |
| Female ^†^ | 1.925 (1.038-3.569) | 1.831 (1.009-3.323) |
| Diabetes mellitus | 0.522 (0.068-4.039) |  |
| Dialysis vintage | 0.998 (0.989-1.008) |  |
| Systolic blood pressure (per 1 mmHg) | 1.01 (0.996-1.024) |  |
| Statin use prior to KT | 0.957 (0.465-1.970) |  |
| Desensitization ^†^ | 1.894 (0.948-3.782) | 2.024 (1.021-4.011) |
| Early statin use ^†^ | 0.364 (0.196-0.677) | 0.378 (0.213-0.671) |
| Deceased donor ^†^ | 2.288 (0.805-6.507) | 2.607 (1.332-5.103) |
| Female donor ^†^ | 1.914 (0.972-3.766) | 1.984 (1.085-3.631) |
| Donor age | 1.010 (0.982-1.040) |  |
| Donor hypertension | 1.358 (0.594-3.105) |  |
| Donor eGFR | 0.999 (0.986-1.012) |  |

aHR, adjusted hazard ratio; cHR, crude hazard ratio; CI, confidence interval; eGFR, estimated glomerular filtration rate; KT, kidney transplant.

†P value <0.05

**Supplement 6.** Kaplan-Meier curves for (A) death-censored graft survival and (B) patient survival by early statin use, extending the initiation time of statin treatment from 1-year to 2-years.

(A)


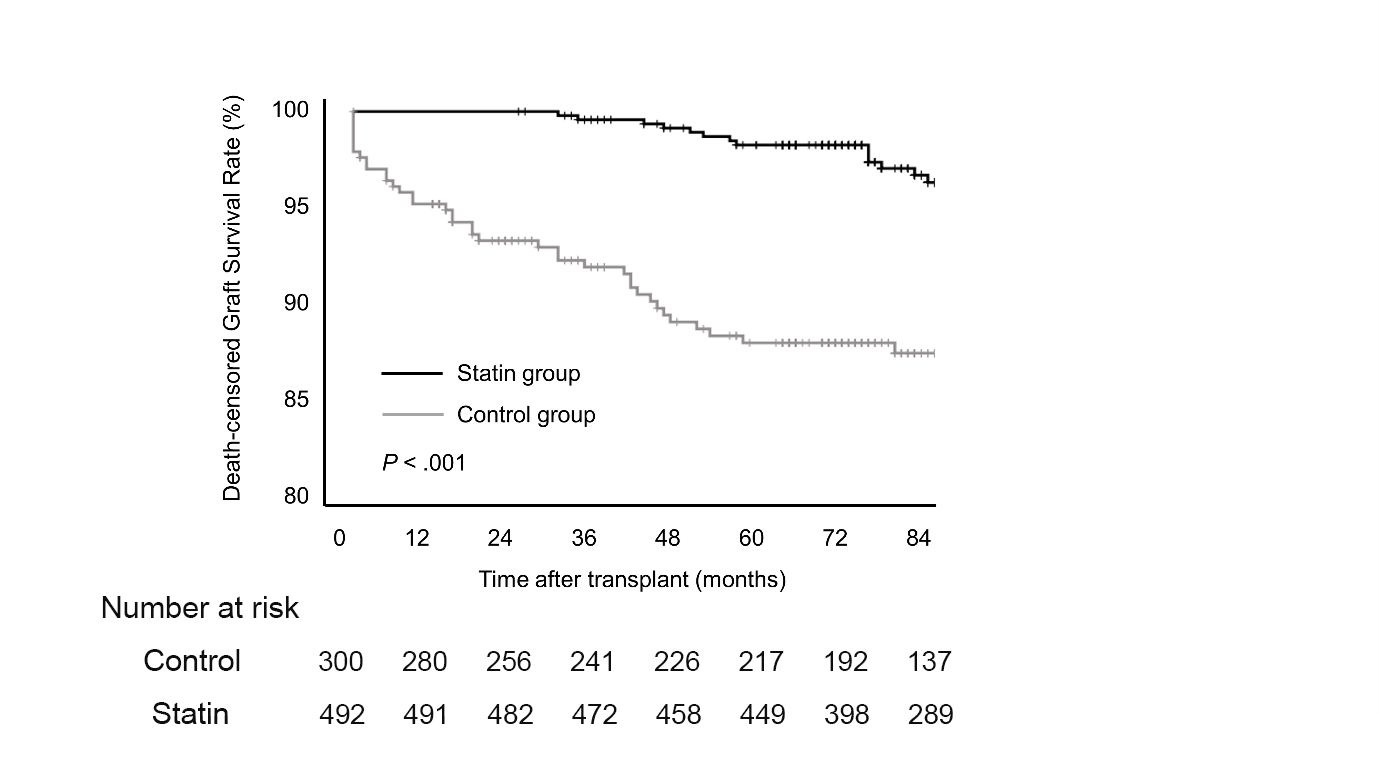


(B)


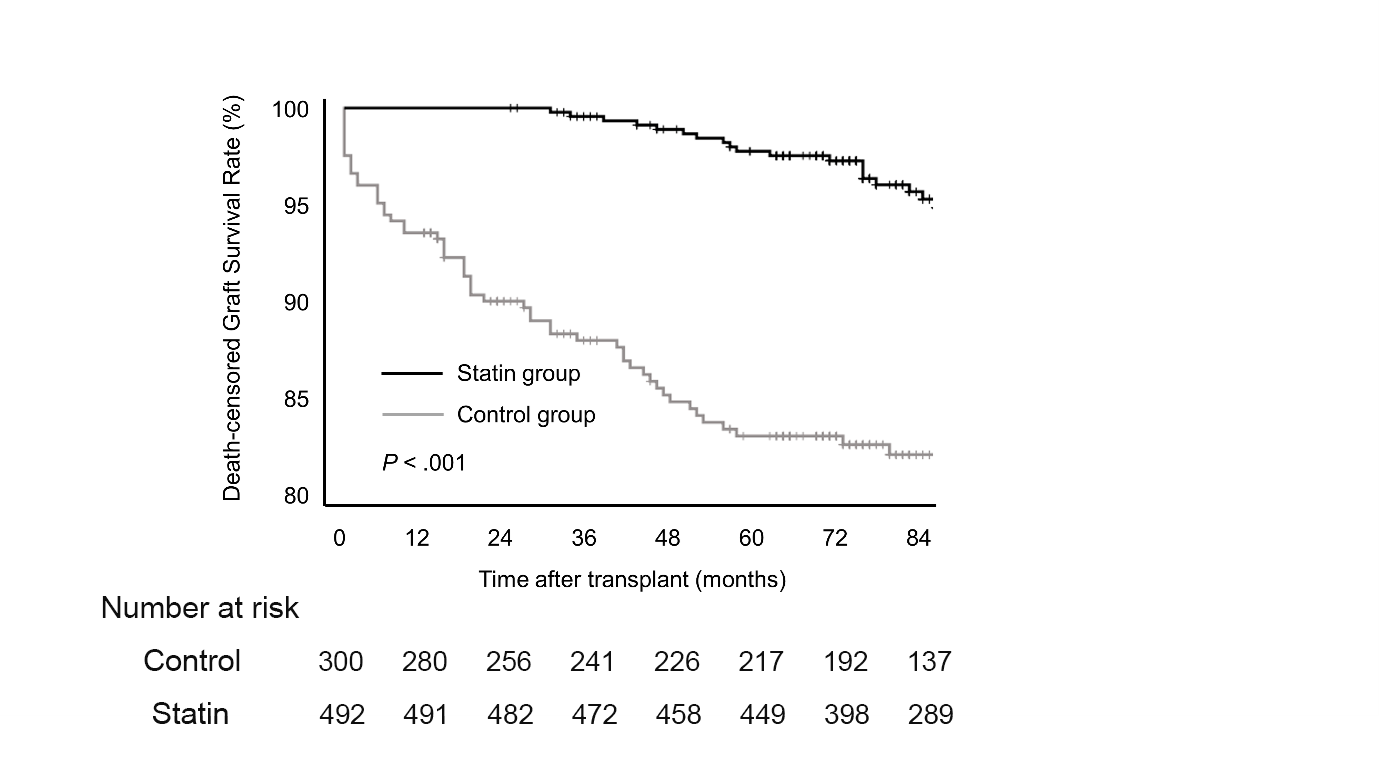


**Supplement 7.** Risk factors for (A) death-censored graft survival and (B) patient survival by early statin use, extending the initiation time of statin treatment from 1-year to 2-years.

**(A)**

| **Factors** | **Univariable**  **cHR (95% CI)** | **Multivariable**  **aHR (95% CI)** |
| --- | --- | --- |
| Recipient age | 1.009 (0.984-1.034) |  |
| Female | 1.432 (0.769-2.666) |  |
| Diabetes mellitus | 1.493 (0.803-2.775) |  |
| Dialysis vintage | 0.995 (0.985-1.004) |  |
| Systolic blood pressure (per 1 mmHg) | 1.008 (0.995-1.021) |  |
| Statin use prior to KT | 1.397 (0.747-2.613) |  |
| Desensitization ^†^ | 2.711 (1.421-5.175) | 2.419 (1.322-4.426) |
| Early statin use ^†^ | 0.191 (0.103-0.356) | 0.234 (0.131-0.419) |
| Deceased donor | 2.706 (0.983-7.446) |  |
| Female donor ^†^ | 2.283 (1.198-4.352) | 2.085 (1.163-3.739) |
| Donor age | 0.996 (0.970-1.022) |  |
| Donor hypertension ^†^ | 2.167 (1.075-4.368) | 2.155 (1.147-4.048) |
| Donor eGFR ^†^ | 0.992 (0.980-1.004) | 0.986 (0.976-0.996) |

aHR, adjusted hazard ratio; cHR, crude hazard ratio; CI, confidence interval; eGFR, estimated glomerular filtration rate; KT, kidney transplant.

†P value <0.05

**(B)**

| **Factors** | **Univariable**  **cHR (95% CI)** | **Multivariable**  **aHR (95% CI)** |
| --- | --- | --- |
| Recipient age ^†^ | 1.020 (0.998-1.043) | 1.018 (0.997-1.040) |
| Female | 1.463 (0.845-2.532) |  |
| Diabetes mellitus ^†^ | 1.726 (1.023-2.912) | 1.706 (1.035-2.813) |
| Dialysis vintage | 0.996 (0.989-1.004) |  |
| Systolic blood pressure (per 1 mmHg) | 1.005 (0.994-1.017) |  |
| Statin use prior to KT | 1.456 (0.849-2.496) |  |
| Desensitization ^†^ | 2.557 (1.46-4.479) | 2.517 (1.447-4.381) |
| Early statin use ^†^ | 0.177 (0.103-0.304) | 0.211 (0.127-0.352) |
| Deceased donor ^†^ | 2.976 (1.273-6.954) | 2.499 (1.415-4.412) |
| Female donor ^†^ | 2.248 (1.275-3.961) | 1.785 (1.102-2.890) |
| Donor age | 0.996 (0.974-1.019) |  |
| Donor hypertension ^†^ | 2.158 (1.173-3.970) | 2.233 (1.300-3.835) |
| Donor eGFR | 0.996 (0.986-1.006) |  |

aHR, adjusted hazard ratio; cHR, crude hazard ratio; CI, confidence interval; eGFR, estimated glomerular filtration rate; KT, kidney transplant.

†P value <0.05

**Supplement 8.** Kaplan-Meier curves for (A) death-censored graft survival and (B) patient survival by early statin use, including patients who discontinued statin therapy during the study period or who initiated statins more than 1-year after transplantation.

A)


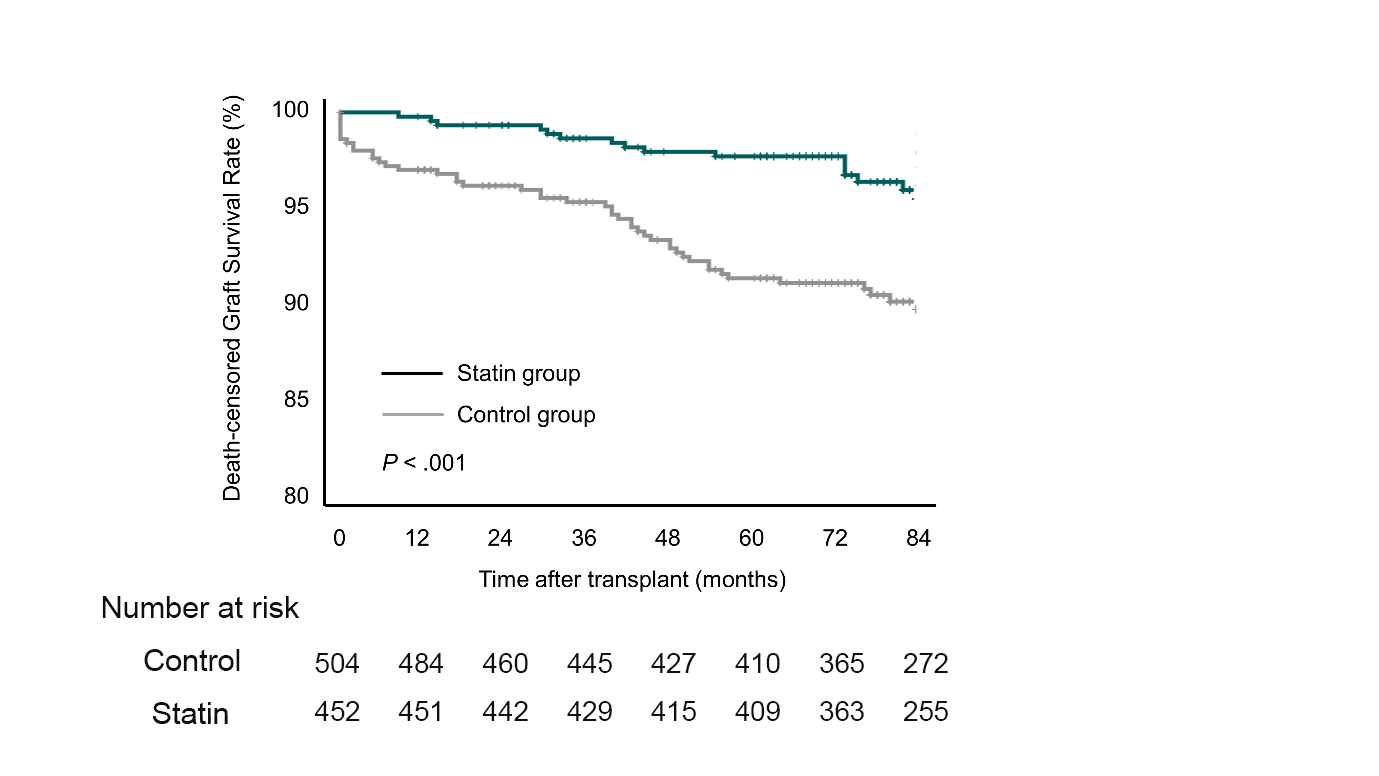


(B)


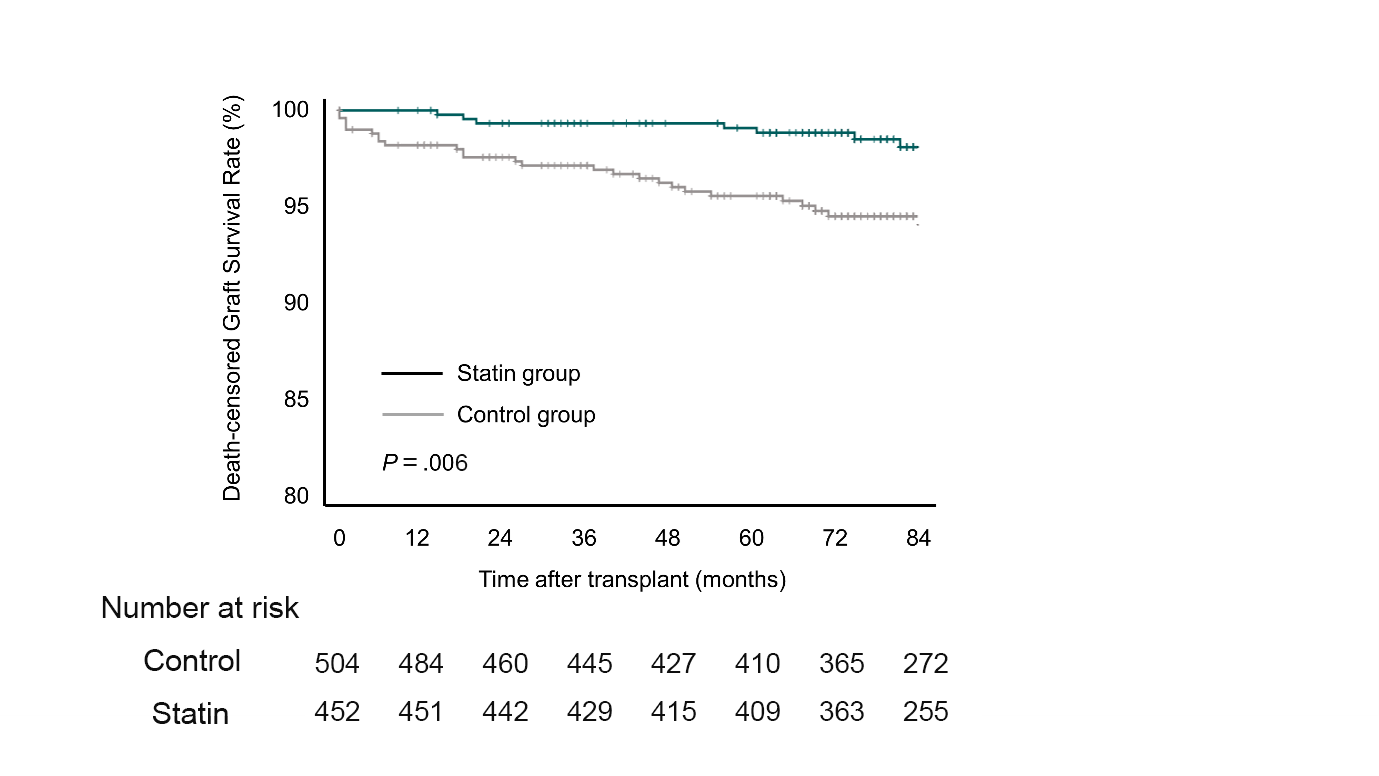


**Supplement 9.** Risk factors for (A) death-censored graft survival and (B) patient survival by early statin use, including patients who discontinued statin therapy during the study period or who initiated statins more than 1-year after transplantation.

**(A)**

| **Factors** | **Univariable**  **cHR (95% CI)** | **Multivariable**  **aHR (95% CI)** |
| --- | --- | --- |
| Recipient age | 1.003 (0.983-1.024) |  |
| Male | 0.819 (0.49-1.37) |  |
| Diabetes mellitus ^†^ | 1.789 (1.093-2.929) | 2.189 (1.251-3.829) |
| Dialysis vintage | 1 (0.995-1.006) |  |
| Systolic blood pressure (per 1 mmHg) | 1.007 (0.995-1.019) |  |
| Statin use prior to KT | 0.894 (0.526-1.518) |  |
| Desensitization ^†^ | 1.429 (0.841-2.428) | 1.91 (1.068-3.415) |
| Early statin use ^†^ | 0.352 (0.203-0.61) | 0.343 (0.195-0.604) |
| Living donor | 0.508 (0.305-0.844) |  |
| Female donor ^†^ | 1.557 (0.958-2.53) | 2.434 (1.375-4.311) |
| Donor age | 1.012 (0.991-1.033) |  |
| Donor hypertension ^†^ | 2.528 (1.475-4.333) | 2.285 (1.192-4.382) |
| Donor eGFR | 0.993 (0.985-1.001) |  |

aHR, adjusted hazard ratio; cHR, crude hazard ratio; CI, confidence interval; eGFR, estimated glomerular filtration rate; KT, kidney transplant.

†P value <0.05

**(B)**

| **Factors** | **Univariable**  **cHR (95% CI)** | **Multivariable**  **aHR (95% CI)** |
| --- | --- | --- |
| Recipient age ^†^ | 1.064 (1.029-1.101) | 1.057 (1.018-1.098) |
| Male | 0.542 (0.246-1.192) |  |
| Diabetes mellitus | 1.805 (0.909-3.584) |  |
| Dialysis vintage | 1.006 (1.001-1.012) |  |
| Systolic blood pressure (per 1 mmHg) | 0.997 (0.981-1.015) |  |
| Statin use prior to KT | 0.799 (0.374-1.704) |  |
| Desensitization | 1.093 (0.496-2.407) |  |
| Early statin use ^†^ | 0.363 (0.170-0.775) | 0.345 (0.152-0.786) |
| Living donor | 0.605 (0.290-1.261) |  |
| Female donor | 1.530 (0.778-3.010) |  |
| Donor age | 1.018 (0.989-1.048) |  |
| Donor hypertension | 2.069 (0.940-4.555) |  |
| Donor eGFR | 1.002 (0.990-1.014) |  |

aHR, adjusted hazard ratio; cHR, crude hazard ratio; CI, confidence interval; eGFR, estimated glomerular filtration rate; KT, kidney transplant.

†P value <0.05

**Supplement 10.** Kaplan-Meier curves for (A) death-censored graft survival and (B) patient survival by early statin use, excluding patients who had the history of MACE prior to KT.

A)


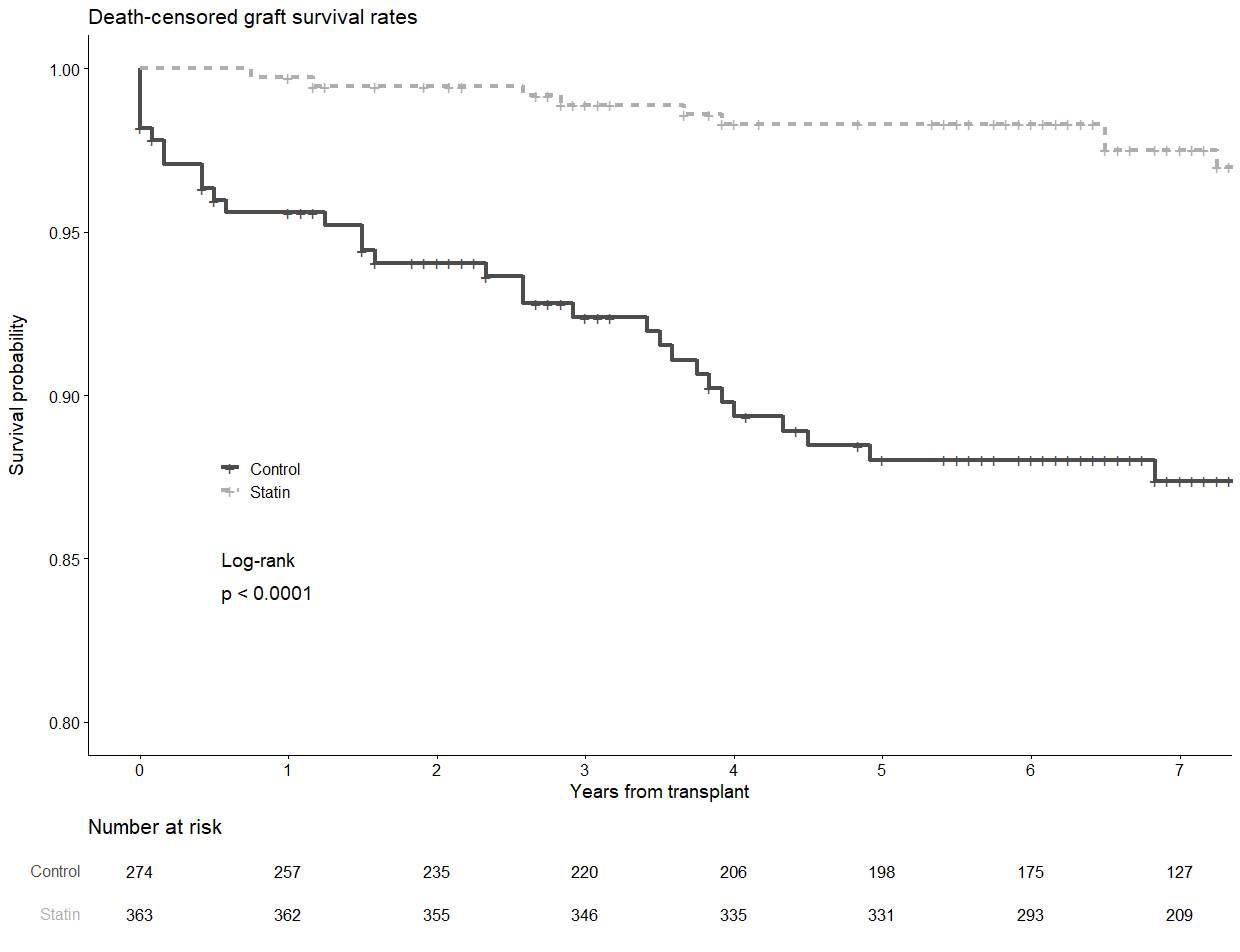


(B)


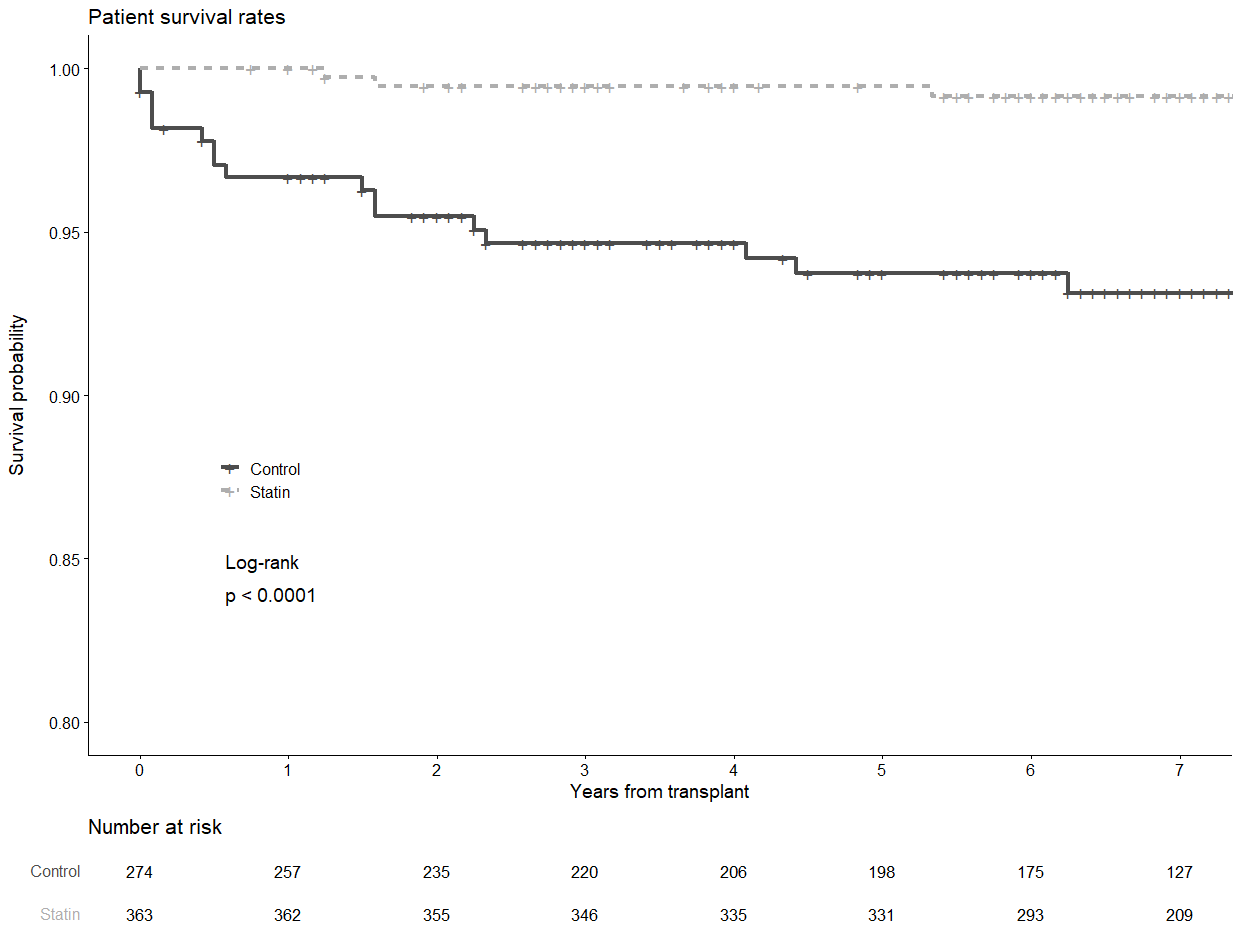


**Supplement 11.** Risk factors for (A) death-censored graft survival and (B) patient survival by early statin use, excluding patients who had the history of MACE prior to KT.

**(A)**

| **Factors** | **Univariable**  **cHR (95% CI)** | **Multivariable**  **aHR (95% CI)** |
| --- | --- | --- |
| Recipient age | 0.995 (0.971-1.020) |  |
| Male | 1.096 (0.595-2.018) |  |
| Diabetes mellitus | 1.230 (0.623-2.429) |  |
| Dialysis vintage | 1.002 (0.996-1.009) |  |
| Systolic blood pressure (per 1 mmHg) | 1.010 (0.995-1.025) | 1.009 (0.994-1.023) |
| Desensitization ^†^ | 1.789 (0.971-3.295) | 2.645 (1.322-5.292) |
| Early statin use ^†^ | 0.213 (0.108-0.420) | 0.214 (0.108-0.427) |
| Living donor | 0.437 (0.232-0.821) | 0.540 (0.218-1.339) |
| Male donor | 0.684 (0.377-1.243) | 0.595 (0.300-1.181) |
| Donor age | 1.023 (0.997-1.050) | 1.002 (0.973-1.032) |
| Donor hypertension | 2.334 (1.156-4.715) | 1.311 (0.558-3.076) |
| Donor eGFR | 0.991 (0.982-1.001) | 0.990 (0.977-1.003) |

aHR, adjusted hazard ratio; cHR, crude hazard ratio; CI, confidence interval; eGFR, estimated glomerular filtration rate; KT, kidney transplant.

†P value <0.05

**(B)**

| **Factors** | **Univariable**  **cHR (95% CI)** | **Multivariable**  **aHR (95% CI)** |
| --- | --- | --- |
| Recipient age ^†^ | 1.06 (1.018-1.105) | 1.063 (1.016-1.113) |
| Male | 2.727 (0.923-8.058) | 1.018 (0.290-3.571) |
| Diabetes mellitus | 1.734 (0.707-4.254) | 1.600 (0.566-4.524) |
| Dialysis vintage | 1.007 (1.000-1.015) | 0.996 (0.983-1.009) |
| Systolic blood pressure (per 1 mmHg) | 0.999 (0.977-1.021) | 0.991 (0.969-1.014) |
| Desensitization ^†^ | 0.198 (0.073-0.536) | 3.642 (1.174-11.300) |
| Early statin use ^†^ | 0.350 (0.147-0.836) | 0.157 (0.049-0.504) |
| Living donor ^†^ | 0.390 (0.152-0.996) | 0.126 (0.026-0.605) |
| Male donor | 1.027 (0.989-1.065) | 0.473 (0.156-1.437) |
| Donor age | 1.780 (0.602-5.260) | 0.999 (0.961-1.037) |
| Donor hypertension | 1.005 (0.989-1.021) |  |
| Donor eGFR | 1.060 (1.018-1.105) |  |

aHR, adjusted hazard ratio; cHR, crude hazard ratio; CI, confidence interval; eGFR, estimated glomerular filtration rate; KT, kidney transplant.

†P value <0.05
